# Immunotherapy in conversion therapy for gastric cancer: current status, progress, and challenges

**DOI:** 10.3389/fonc.2025.1637657

**Published:** 2025-09-22

**Authors:** Yi Su, Xin Zhang, Wei Deng

**Affiliations:** 1 Department of Digestive Oncology, Beijing Chaoyang Integrative Medicine Rescue and First Aid Hospital, Beijing, China; 2 General Surgery Department, Friendship Hospital, Capital Medical University, Beijing, China

**Keywords:** gastric cancer, immunotherapy, conversion therapy, R0 resection rate, multimodal therapy

## Abstract

Gastric cancer remains a global health burden due to its late diagnosis and poor prognosis. Conversion therapy aims to make the initially unresectable tumor resectable through systemic treatment, providing the opportunity for long-term survival. The rise of immunotherapy has brought new potential to this field. Immunotherapy combined with chemotherapy, anti-angiogenic drugs or chemoradiotherapy has shown good efficacy in specific patients. This review summarizes the current evidence of conversion strategies based on immunotherapy, emphasizes key biomarkers, and explores the future direction of precise, multi-modal treatment.

## 1 Introduction

Gastric cancer remains one of the most prevalent and lethal malignancies globally, with high incidence, low early detection rates, and poor survival outcomes. According to GLOBOCAN 2020, gastric cancer ranks fifth in incidence and fourth in cancer-related mortality worldwide, with approximately 1.09 million new cases and 769,000 deaths annually (1, 2). Nearly half of these cases occur in China, where the early detection rate remains below 30%, resulting in 60%-70% of patients being diagnosed at a locally advanced (stage III) or metastatic (stage IV) stage. Correspondingly, five-year survival drops to ~30% for stage III and below 10% for stage IV disease. Due to the lack of early symptoms and effective screening programs, most patients are diagnosed with advanced-stage disease (3, 4).Traditional surgical resection and monotherapy chemotherapy are insufficient to meet the clinical needs of patients with advanced disease. As such, there is growing interest in multidisciplinary strategies aimed at converting initially unresectable tumors into resectable ones.

The emergence of immune checkpoint inhibitors (ICIs) has brought significant advances in the treatment of advanced gastric cancer and opened new avenues for conversion therapy. This review outlines recent progress in immunotherapy-based conversion strategies, summarizes key clinical evidence, and discusses ongoing challenges and future directions in this evolving field (5, 6).

## 2 Concept, indications, and significance of conversion therapy

Although neoadjuvant therapy, systemic therapy, and conversion therapy may utilize similar pharmacological agents, there are fundamental differences in their clinical objectives and the populations to which they apply. Neoadjuvant therapy is primarily administered to patients who are technically resectable, with the aim of reducing tumor volume, enhancing surgical resection rates, and improving long-term survival outcomes. In contrast, systemic therapy is indicated for patients with distant metastases, focusing on prolonging survival and enhancing quality of life.

Conversion therapy occupies a position between these two approaches; it is appropriate for patients initially deemed either technically or oncologically unresectable or marginally resectable (6, 7). The primary objective of conversion therapy is to achieve R0 resection following systemic treatment—such as chemotherapy, targeted therapies, or immunotherapy—thereby improving prognosis (8). Clinical studies have demonstrated that the median overall survival (OS) for patients who successfully undergo R0 resection can reach 24–36 months, significantly surpassing the 8–12 months observed in patients receiving palliative care (9–13).

Candidates for conversion therapy can generally be categorized into two groups: (i)patients with locally advanced disease (e.g., T4b or N2-N3), where invasion of adjacent structures or bulky nodal metastases renders upfront resection unfeasible; and (ii)Patients with favorable tumor biology (such as her2 positive or MSI-H/dMMR) or limited metastasis (single-organ metastasis such as liver metastasis, ovarian metastasis, retroperitoneal lymph node metastasis, supraclavicular lymph node metastasis, etc.) may be resectable after systemic treatment (10, 14).

To optimize patient selection, a biologically oriented classification system for stage IV gastric cancer has been proposed, integrating tumor burden, resectability, and peritoneal dissemination status. This framework divides patients into four categories:

Category 1: Technically resectable metastases without macroscopic peritoneal dissemination, such as solitary liver lesions, isolated para-aortic lymph node metastasis, or positive peritoneal cytology. These patients are typically treated with neoadjuvant chemotherapy rather than conversion therapy.
Category 2: Marginally resectable metastases, including multiple liver lesions, major vascular involvement, or extensive nodal disease. These cases may benefit from systemic therapy to enable potential resection.
Category 3: Includes patients with peritoneal metastasis, which is traditionally associated with poor prognosis and limited treatment options. However, in highly selected cases, systemic therapy may induce a good peritoneal response, making it possible to consider cytoreductive surgery in specialized centers.
Category 4: Non-curable metastases, such as peritoneal spread with distant organ involvement (e.g., lung, bone), where conversion therapy is only considered in highly responsive tumors (15).

This classification framework helps distinguish patients eligible for surgery with or without induction therapy and supports individualized treatment planning. As an integrated, multidisciplinary strategy, conversion therapy represents a major paradigm shift in the management of advanced gastric cancer—from empirical, stage-based approaches toward personalized, biology-guided treatment. Future studies are needed to enhance patient stratification, refine systemic regimens, and improve the R0 resection rate.

## 3 Application and progress of immunotherapy in conversion therapy

### 3.1 Immune checkpoint inhibitors combined with chemotherapy

Building upon prior progress in perioperative chemotherapy, immune checkpoint blockade has emerged as a promising strategy to further enhance resectability and long-term survival in gastric cancer. Pivotal trials such as MAGIC and FLOT4-AIO established the value of neoadjuvant chemotherapy followed by surgery, demonstrating improved tumor downstaging (e.g., tripling ypT0 rates), a 15%-20% increase in R0 resection rates, and prolonged 5-year survival, without increasing perioperative morbidity (16–18).These findings established neoadjuvant chemotherapy as the standard backbone for locally advanced, resectable gastric cancer, and laid the foundation for subsequent integration of immunotherapy in the conversion setting.

Multiple phase III trials have since confirmed that chemo-immunotherapy confers a survival advantage as first-line treatment for advanced gastric cancer. These findings have not only expanded the therapeutic scope of immune checkpoint inhibitors but also laid the foundation for their earlier incorporation into the management of locally advanced gastric cancer(LAGC). In Asian populations, the phase III ORIENT-16 trial demonstrated that sintilimab plus chemotherapy significantly prolonged OS compared with chemotherapy alone—in the overall population (15.2 vs. 12.3 months; HR = 0.77, P = 0.009) and particularly in patients with PD-L1 CPS ≥5 (19.2 vs. 12.9 months; HR = 0.66, P < 0.001)—supporting its use as a first-line treatment for advanced gastric or gastroesophageal junction adenocarcinoma in Chinese patients (19).Similarly, in the Chinese subgroup of CheckMate-649, 5-year OS reached 24% versus 8% with chemotherapy alone in CPS≥5 patients (20, 21). KEYNOTE-062 has showed that pembrolizumab monotherapy has achieved an mOS of 17.4 months in patients with CPS ≥10, outperforming chemotherapy (10.8 months) (22).More broadly, KEYNOTE-859 confirmed the survival benefit of pembrolizumab plus chemotherapy in an unselected advanced GC/GEJC population (mOS: 13.0 vs 11.3 months, HR = 0.78, P < 0.001). In the Chinese subgroup, mOS improved to 15.9 months vs 12.2 months (HR = 0.68), and reached 21.4 months in patients with CPS≥10 (HR = 0.51), with an ORR of 80%. These findings suggest that higher PD-L1 expression may be associated with greater clinical benefit. Collectively, these findings indicate that a subset of PD-L1-high patients may already have entered a “long-term survival plateau”, and that immune therapy is moving beyond palliative intent toward earlier-stage disease (23).

Building on these advances, the PD-1/CTLA-4 bispecific antibody cadonilimab exhibited broad and sustained efficacy in the COMPASSION-15 trial (24). Notably, nearly half of the enrolled patients had a PD-L1 CPS <5, and 23% had CPS <1. Interim analysis showed that cadonilimab combined with chemotherapy significantly improved OS compared to chemotherapy alone (15.0 vs 10.8 months; HR = 0.62; P < 0.001). Even among patients with CPS <5, OS was extended to 14.8 months vs 11.1 months (HR = 0.70), challenging the assumption that PD-L1-low tumors are unresponsive to immunotherapy. These findings suggest that dual checkpoint inhibition may achieve consistent efficacy across a broader biomarker spectrum.

Against the backdrop of survival benefits achieved with immunotherapy in advanced gastric cancer, several pivotal phase III trials have explored its application in earlier stages, particularly in patients with borderline resectable or locally advanced disease. Although these studies are largely designed as perioperative trials, many enrolled populations that reflect typical conversion therapy scenarios—namely, tumors initially deemed unresectable or marginally operable. As such, these findings have important implications for immunotherapy-driven conversion strategies aiming to enhance resectability and long-term outcomes.

The global phase III MATTERHORN trial (NCT04592913) is a randomized, double-blind, placebo-controlled study assessing durvalumab in combination with FLOT chemotherapy in patients with resectable GC/GEJC (25). A total of 474 patients with clinical stage T2-T4 and N0-3M0 disease were randomized to receive perioperative FLOT with or without durvalumab, followed by 10 cycles of adjuvant immunotherapy. Interim results showed a significantly higher pCR rate in the durvalumab arm (19% vs 7%; odds ratio = 3.08; P < 0.00001), along with superior tumor downstaging (pT0: 21% vs 10%; pN0: 47% vs 33%). R0 resection and surgical completion rates were comparable between groups. These findings support the potential of integrating immunotherapy into perioperative regimens to enhance pathological response without compromising surgical safety. Long-term survival data are pending.KEYNOTE-585 (NCT03221426) is the first global phase III trial to evaluate perioperative PD-1 blockade in combination with chemotherapy for resectable GC/GEJC. Patients with cT3-T4/N+M0 disease received three preoperative and eleven postoperative cycles of chemotherapy, along with pembrolizumab or placebo. The pembrolizumab group achieved a significantly higher pCR rate (13.4% vs 2.0%; P < 0.001) and extended median event-free survival (EFS: 44.4 vs 25.7 months; HR = 0.81). Although the difference in OS was not statistically significant (71.8 vs 55.7 months; HR = 0.86; P = 0.057), a stronger benefit was observed in patients with PD-L1 CPS ≥1 (HR = 0.73). Treatment-related adverse events (TRAEs) were comparable between groups (64% vs 63%). These results support the feasibility of perioperative chemo-immunotherapy and highlight the potential role of extended adjuvant immunotherapy in high-risk populations (26, 27).

Acknowledging regional variations in tumor biology and treatment response, two pivotal Chinese phase II studies-NEOSUMMIT (28) and PERSIST (29)-have provided key evidence supporting immunotherapy-based conversion strategies in East Asian populations. The NEOSUMMIT trial (NCT04354662) enrolled 108 patients with locally advanced GC/GEJC and randomized them to receive toripalimab plus SOX/XELOX or chemotherapy alone. The immunotherapy arm demonstrated significantly higher rates of tumor regression grade (TRG) 0/1 (44.4% vs 20.4%; P = 0.009) and pCR: 22.2% vs 7.4%; P = 0.030). Notably, all six patients in the dMMR subgroup achieved pCR, compared to none in the control group. Tumor downstaging (ypT0-2) occurred more frequently with toripalimab (46.3% vs 22.2%; P = 0.008). Patients with intestinal or mixed histological types exhibited better responses than those with diffuse-type tumors (2.3-fold higher, P < 0.01). Adverse event rates were comparable (TRAEs: 37.0% vs 33.3%), and immune-related toxicities remained manageable. The PERSIST trial (29) (NCT04982939) adopted a “sandwich” strategy-neoadjuvant immunotherapy, surgery, and adjuvant immunotherapy-using sintilimab combined with SOX in 240 patients with locally advanced GC/GEJC. The combination group achieved a pCR rate of 27.9%, significantly higher than 4.8% in the control arm (P < 0.001), and a major pathological response (MPR,≤10% residual tumor) rate of 65.2% versus 20.4%. Tumor downstaging was observed in 79.7% of patients, and the R0 resection rate exceeded 91% (91.8% vs 89.3%). Grade 3–4 TRAEs occurred in only 4.1% of cases, and no perioperative mortality was reported. As the first phase II trial to validate a domestically developed PD-1 inhibitor in this context, PERSIST strongly supports the feasibility and safety of conversion immunotherapy in Chinese patients. Together with NEOSUMMIT, it highlights the potential for enhanced immunotherapeutic sensitivity in East Asian populations, possibly due to higher prevalence of proximal tumors, Epstein-Barr virus (EBV) positivity, and favorable molecular subtypes such as dMMR/MSI-H and intestinal histology. **Table 1** lists an overview of key clinical trials investigating chemoimmunotherapy in conversion therapy.

**Table 1:** Key clinical trials of immunotherapy combined with chemotherapy in conversion therapy for gastric cancer.

Study	Phase	Population	Treatment	Main outcomes	Remarks (subgroup analysis)
(19) ORIENT-16	Ill	Advanced GC/GEJC	Sintilimab + Chemotherapy vs Chemotherapy	mOS:15.2 vs 12.3 months in overall population ORR: 58.2%	Greater benefit in PD-L1 CPS ≥5 subgroup; (mOS: 19.2 vs 12.9 months)
(20) CheckMate-649	Ill	Advanced GC/GEJC	Nivolumab + Chemotherapy vs Chemotherapy	5-year OS rate:24% vs 8% (PD-L1 CPS ≥5); prolonged mOS	Greater benefit in PD-L1 CPS ≥5 subgroup ORR: 68% vs 48%
(22) KEYNOTE-062	Ill	Advanced GC (PD- L1CPS ≥1 or ≥10)	Pembrolizumab vs Chemotherapy	mOS:17.4 vs 10.8 months in PD-L1 CPS ≥10 subgroup	Monotherapy favored in PD-L1 CPS ≥1Q subgroup
(23) KEYNOTE-859	Ill	HER2-negative advanced GC	Pembrolizumab + Chemotherapy vs Chemotherapy	mOS:13.0 vs 11.5 months; HR=0.78	Benefit seen across all PD-L1 CPS groups; more pronounced in CPS ≥10
(28) NEOSUMMIT	II	Locally advanced GC	Toripalimab +Chemotherapy vs Chemotherapy	Significantly higher pCR rate, especially in dMMR and intestinal subgroups	dMMR subgroup achieved ~50% pCR
(29) PERSIST	II	Locally advanced GC/GEJC	Sintilimab + SOX vs SOX	pCR rate 27.9%; RO rate: 95%	Higher pCR in PD-L1 CPS ≥5 subgroup

### 3.2 Multimodal combination therapy strategy

**Figure 1:**
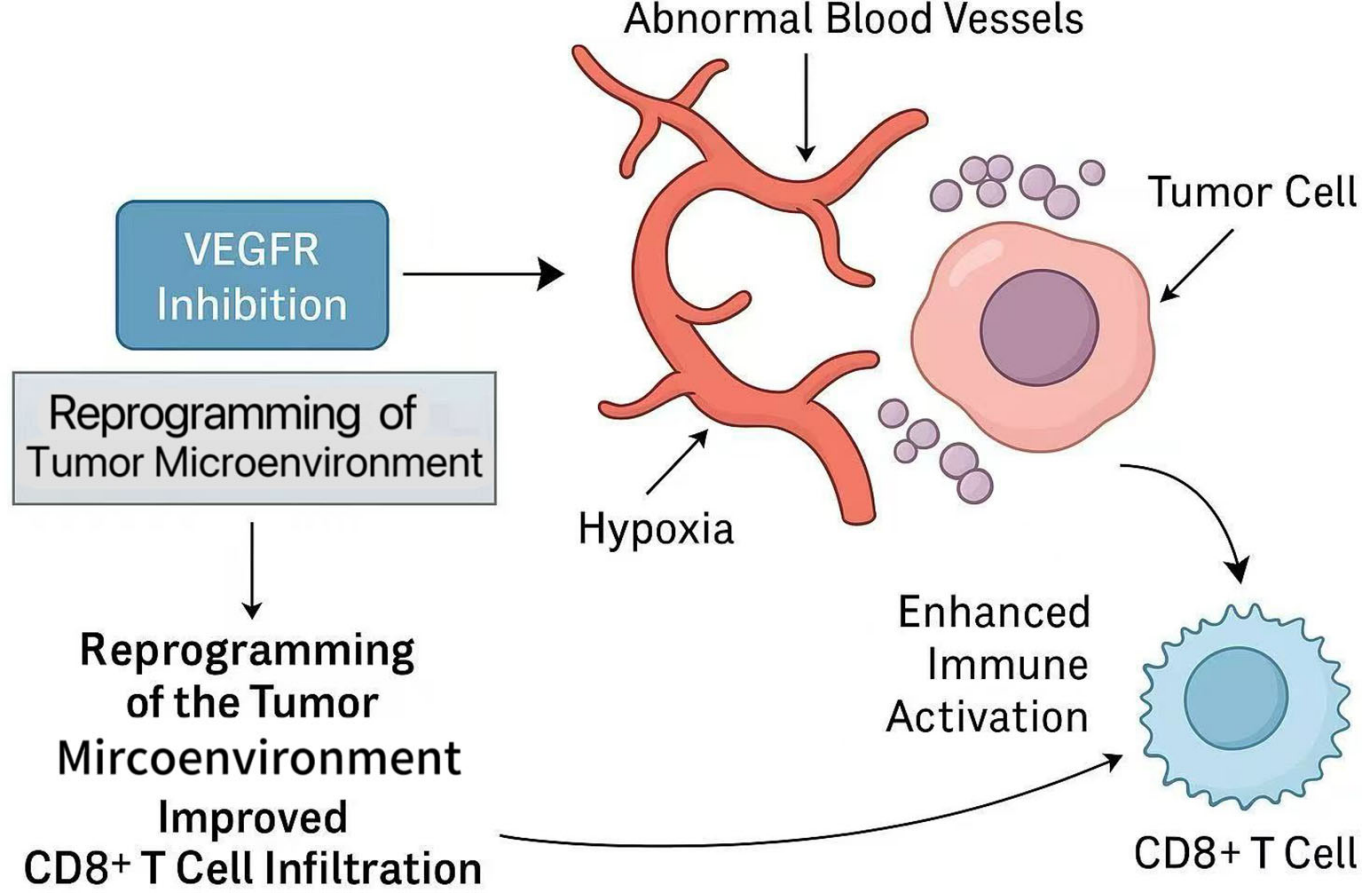
Mechanism by which VEGFR inhibition enhances anti-tumor immunity and synergizes with immune checkpoint blockade.

Beyond chemical immunotherapy, multimodal strategies combining immune checkpoint inhibitors, anti-angiogenic drugs, and chemotherapy have shown encouraging potential in increasing conversion rates. Antiangiogenic therapy enhances this synergy by normalizing the tumor vascular system, alleviating hypoxia, and reprogramming the tumor microenvironment (TME). These changes promote CD8^+^ T cell infiltration and effector function, while immune checkpoint blockade restores T cell activity (30, 31). These mechanisms jointly drive the synergistic anti-tumor immune response, as shown in **Figure 1**.

The DRAGON-IV/AHEAD-G208 (32)trial, a phase III multicenter randomized controlled study, evaluated the efficacy of triple therapy with camrelizumab, apatinib, and SOX chemotherapy in patients with resectable gastric cancer or GEJC. The combination arm achieved a significantly higher pCR rate (18.3% vs 5.0%) compared to chemotherapy alone. While surgical completion and R0 resection rates were similar between groups, the triple regimen induced deeper pathological responses. Biomarker analysis showed a 68% reduction in VEGF pathway activity, supporting the hypothesis that anti-angiogenic therapy facilitates TME remodeling and immunotherapy synergy. In terms of safety, grade ≥ 3 TRAEs-primarily neutropenia and thrombocytopenia-were manageable and did not impair surgical feasibility. Importantly, biomarker analyses indicated that patients with PD-L1 CPS ≥ 5 and EBV-positive tumors achieved higher pCR rates, underscoring the value of biomarker-guided patient selection in optimizing treatment outcomes.

Collectively, these findings support multimodal immunotherapy as a viable strategy for conversion therapy, particularly in biomarker-enriched subgroups.

### 3.3 Cutting-edge exploration of dual immunotherapy

While bispecific antibodies like cadonilimab have broadened immunotherapy applicability in PD-L1-low populations, dual immune checkpoint blockade offers a chemotherapy-free alternative for molecularly defined subgroups-particularly patients with MSI-H or dMMR gastric cancer. This approach marks a paradigm shift from histopathologic staging to molecular-driven treatment selection, offering a strong rationale for conversion therapy in patients with high tumor immunogenicity but initially unresectable or borderline-resectable disease.

The INFINITY trial (NCT04817826), a phase II study, evaluated neoadjuvant durvalumab (anti-PD-L1) plus tremelimumab (anti-CTLA-4) in patients with resectable MSI-H gastric or gastroesophageal junction adenocarcinoma. The study reported a pCR rate of 60% and a 2-year relapse-free survival (RFS) rate of 85% (33). Subgroup analysis revealed that patients with T2-T3 tumors achieved a pCR rate of 88.9%, while those with T4 disease showed a markedly lower rate of 16.7%, suggesting an inverse correlation between tumor invasiveness and immunotherapy responsiveness. The NEONIPIGA trial (NCT04006262) investigated neoadjuvant nivolumab plus ipilimumab followed by adjuvant nivolumab in patients with localized MSI-H/dMMR gastric or gastroesophageal junction adenocarcinoma. The study achieved a pCR rate of 58.6% and a major pathological response (MPR, ≤10% residual tumor) rate of 79% (34). Immune-related adverse events (irAEs) primarily colitis, pneumonitis, and hepatitis were manageable and did not impair surgical feasibility or R0 resection outcomes.

Collectively, these studies suggest that dual checkpoint inhibition can induce profound tumor regression in MSI-H/dMMR gastric cancer, enabling curative resection without chemotherapy. This chemo-free strategy may be particularly valuable for patients who are medically unfit for cytotoxic agents or exhibit high immunogenicity. However, broader clinical adoption requires confirmation in randomized phase III trials with stratification by T stage, baseline resectability, and molecular features.

### 3.4 New attempts at immuno-combination radiotherapy

Radiotherapy has historically played a limited role in gastric cancer management. However, emerging studies combining radiotherapy with immunotherapy have introduced new strategies for improving local tumor control and resectability in patients with locally advanced, borderline resectable, or initially unresectable disease. The synergistic effect arises from radiotherapy’s capacity to induce immunogenic cell death, enhance antigen presentation, and remodel TME, thereby increasing T cell infiltration and potentiating immune checkpoint blockade.

The SHARED study (35) (ChiCTR1900024428) evaluated neoadjuvant chemoradiotherapy combined with sintilimab in 34 patients with cT3-4N+ or T4b gastric/gastroesophageal junction adenocarcinoma. Patients received concurrent radiotherapy (45-50.4 Gy), chemotherapy, and PD-1 blockade. The trial reported a pCR rate of 38.2% (13/34; 95% CI, 22.2%-56.4%), substantially higher than historical pCR rates for conventional chemoradiotherapy (10%-15%) (36–38). The R0 resection rate reached 94.7%, and median EFS was 21.1 months. These results suggest that immuno-radiotherapy may enhance pathological response and improve curative resection rates in patients with high-risk locoregional disease.

The Neo-PLANET trial (39) enrolled 36 patients with cT3-4N+ proximal gastric cancer and assessed camrelizumab combined with concurrent chemoradiotherapy (45 Gy). The pCR rate reached 33.3% (12/36; 95% CI, 18.6%-51.0%), slightly lower than in the SHARED study. However, nodal downstaging (ypN0) was achieved in 77.8% of patients, and the 2-year OS rate reached 76.1%. These findings suggest that, in addition to enhancing pCR, radioimmunotherapy may offer substantial regional disease.

Safety profiles varied between the two regimens. In SHARED, grade 3–4 TRAEs occurred in 39.3%, primarily hematologic toxicities. In contrast, the Neo-PLANET trial reported a higher TRAE rate of 80.6%, largely attributable to lymphopenia, though without compromising surgical feasibility. Both studies achieved high R0 resection rates (91.7%-94.7%), exceeding the typical 85%-89% observed with conventional chemoradiotherapy (40).

Overall, early-phase data support the feasibility of combining immunotherapy with chemoradiotherapy in locally advanced gastric cancer. Nevertheless, validation in larger randomized trials is warranted to confirm survival benefits and optimize treatment protocols. **Table 2** summarizes representative multimodal and emerging conversion therapy strategies in combination with immunotherapy.

**Table 2:** Key clinical trials of multimodal and emerging immunotherapy strategies in conversion therapy for gastric cancer.

Study	Phase	Population	Treatment	Main outcomes	Remarks (subgroup analysis)
DRAGON-IV/ (32) AHEAD-G208	Ill	Resectable GC/GEJC	Camrelizumab + Apatinib +SOX VS SOX	pCR:18.3% vs 5.0%; VEGF pathway inhibition:68%	Higher pCR in PD-L1 CPS ≥5 and EBV- positive patients
(33) INFINITY	II	MSI-H GC/GEJC	Durvalumab + Tremelimumab	pCR: 60%;2-year RFS:85%	pCR: 88.9%in T2-T3; only 16.7% in T4 tumors
(34) NEONIPIGA	II	MSI-H GC/GEJC	Nivolumab + lpilimumab (neoadjuvant) + Nivolumab (adjuvant)	pCR: 58.6%;MPR: 79%	Highly effective in dMMR patients
(35) SHARED	II	Locally advanced GC	Sindilizumab + Radiotherapy	pCR: 38.2%;RO: 94.7%; mEFS 21.1 months	Achieved pCR >30%, considered breakthrough
(39) Neo-PLANET	II	Locally advanced GC/GEJC	Camrelizumab +Radiotherapy	pCR: 33.3%;ypNO: 77.8%; RO:91.7%	Grade 3-41ymphocytopenia: 75% but feasible surgery

## 4 Discussion

Despite encouraging progress, the application of immunotherapy in the conversion therapy of LAGC is still in its infancy and is developing rapidly. Many key challenges still need to be addressed before the immune-based treatment strategies are widely incorporated into clinical practice.

First, there is a disconnect between pathological response and long-term survival. For example, although the KEYNOTE-585 trial has shown significant improvements in pCR and EFS, it has not yet translated into statistically significant OS benefits, highlighting the urgent need for reliable alternative endpoints and long-term follow-up.

Secondly, there is a high degree of heterogeneity in immune responses between patients. Patients with diffuse histological subtypes or low PD-L1 expression levels often have limited benefits; in addition, compared with advanced diseases, the predictive value of traditional biomarkers (such as PD-L1 combined positive score, CPS) in perioperative period is relatively weak.

Third, irAEs-such as pneumonia, hepatitis, and colitis-although mostly manageable, still cause about 10%-15% of patients to terminate treatment early, potentially affecting the integrity of treatment options in the real world (41–43).

In addition, the biomarker system is not yet uniform, which continues to limit the accuracy of patient screening. Although PD-L1 CPS, microsatellite instability (MSI) and tumor mutation burden (TMB) have predictive value in specific populations, their roles in perioperative treatment decisions are still inconsistent and lack prospective verification (44). In recent years, the predictive value of inflammation-related indicators has gradually attracted attention. Systemic inflammatory response indicators such as neutrophil-to-lymphocyte ratio (NLR) and platelet-to-lymphocyte ratio (PLR) have not only been shown to be associated with pathological remission rate and OS in a number of studies, but also been found to be significantly associated with R0 resection rate in a retrospective cohort study in Slovakia, suggesting that they have potential application value in efficacy prediction and risk stratification in immune combined conversion therapy (45).

## 5 Conclusion

Immunotherapy has opened up a new way for the conversion therapy of LAGC, but its clinical integration is still limited by biological complexity, regional variability and lack of effective predictors. In the future, the implementation of dynamic monitoring tools such as ct DNA and radiomics, as well as multi-dimensional biomarker analysis that integrates genomic, immunological and microbiome data, is expected to improve patient selection. Moreover, next-generation immunotherapy platforms - including bispecific antibodies, antibody-drug conjugates (ADCs), neoantigen vaccines, and oncolytic viruses -may improve efficacy while minimizing systemic toxicity (46). These innovations will help realize the full potential of immunotherapy-based transformation strategies in clinical practice.

## Glossary

GC: Gastric cancer
R0 resection: Curative resection with negative margins
pCR: Pathological complete response
ICI: Immune checkpoint inhibitor
OS: Overall survival
MSI-H: Microsatellite instability–high
dMMR: Deficient mismatch repair
LAGC: Locally advanced gastric cancer
CPS: Combined positive score
PD-1: Programmed cell death protein 1
PD-L1: Programmed death-ligand 1
GEJC: Gastroesophageal junction cancer
CTLA-4: Cytotoxic T-lymphocyte–associated protein 4
EFS: Event-free survival
TRAEs: Treatment-related adverse events
MPR: Major pathological response
EBV: Epstein–Barr virus
TME: Tumor microenvironment
CTLA-4: Cytotoxic T-lymphocyte–associated protein 4
RFS: Relapse-free survival
ctDNA: Circulating tumor DNA
ADC: Antibody–drug conjugate.
