# Correction: Immunotherapy in conversion therapy for gastric cancer: current status, progress, and challenges

**DOI:** 10.3389/fonc.2025.1730862

**Published:** 2025-11-20

**Authors:** Yi Su, Xin Zhang, Wei Deng

**Affiliations:** 1 Department of Digestive Oncology, Beijing Chaoyang Integrative Medicine Rescue and First Aid Hospital, Beijing, China; 2 General Surgery Department, Friendship Hospital, Capital Medical University, Beijing, China

**Keywords:** gastric cancer, immunotherapy, conversion therapy, R0 resection rate, multimodal therapy

Author “Yi Su” was erroneously assigned as the corresponding author in the published article. The correct corresponding author is “Wei Deng”.

The original version of this article has been updated.

